# Lipid Nanoparticle Development for A Fluvid mRNA Vaccine Targeting Seasonal Influenza and SARS-CoV-2

**DOI:** 10.1038/s41541-025-01153-6

**Published:** 2025-06-11

**Authors:** Jiin Felgner, Jenny E. Hernandez-Davies, Erwin Strahsburger, Emily Silzel, Rie Nakajima, Aarti Jain, Jacob Laster, Jui-Lin Chiang, Yali Tsai, Philip L. Felgner, D. Huw Davies, Li Liang

**Affiliations:** 1 Vaccine Research and Development Center, Department of Physiology and Biophysics, University of California Irvine, Irvine, CA USA; 2 Polaris Pharmaceuticals, LLC, San Diego, CA USA

**Keywords:** RNA vaccines, Influenza virus, SARS-CoV-2

## Abstract

mRNA vaccines represent a promising alternative to conventional vaccines, as demonstrated by the rapid deployment of mRNA vaccines during the recent COVID-19 pandemic. In this work, we have adapted and fine-tuned various reported mRNA lipid nanoparticle (LNP) synthesis and preparation procedures, evaluated a range of ionizable cationic lipids, and identified top-performing LNP formulations. The impact of uridine modification on mRNA’s ability to trigger immune responses has also been explored. Our findings indicate that both unmodified mRNA and N1-methyl pseudouridine-modified mRNA successfully induced an antigen-specific antibody response in mice, while the methoxy uridine-modified mRNA did not. Based on these studies, we constructed a bivalent Fluvid mRNA vaccine, consisting of LNPs encapsulating uridine-unmodified mRNA encoding either a transmembrane domain-deleted hemagglutinin or the full-length native spike protein. This vaccine stimulated robust T cell and B cell immune responses and conferred 100% protective efficacy against challenge with either influenza or SARS-CoV-2 viruses in the mouse model, without compromising efficacy compared to administering each monovalent vaccine individually. Our data suggest that the multivalent mRNA vaccine can offer protection against different viruses by generating humoral and cellular responses against multiple antigens at the same time.

## Introduction

Multivalent vaccines have been a widely-used strategy for combating multiple pandemic or seasonal diseases^1,2^. Currently broad protection against seasonal influenza is achieved by using administration of a multivalent (tri- or quadrivalent) vaccine consisting of two inactivated influenza A virus strains—H1N1 and H3N2, with one or two influenza B viruses. It is also anticipated that as SARS-CoV-2 continues to evolve and adapt to human populations, several variants will remain endemic and circulate simultaneously, leading to a need for seasonal COVID-19 vaccination. Moreover, it is likely that influenza virus may become the next pandemic as it has caused four major pandemics in the last century: the 1918 H1N1 Spanish flu, the 1957 H2N2 Asian flu, the 1968 H3N2 Hong Kong flu, and the 2009 H1N1 swine flu pandemic. Therefore, combining the vaccines against seasonal or pandemic COVID-19 and influenza into a multivalent vaccine may be a practical modality to improve patient compliance and reduce costs^3–5^. As with influenza, there is concern that SARS-CoV-2 variants that escape immunity from vaccination or natural exposure will emerge, which will reduce efficacy of existing vaccines and necessitate new vaccines derived from the variants^6,7^. The mRNA platform may be able to help address this by virtue of its agility and potential for rapid deployment.

The origins of mRNA vaccines can be traced to pioneering gene therapy studies performed over 30 years ago in which RNA was transfected into cells using neutral^8,9^ or cationic liposomes^10,11^, in much the same way as an enveloped virus infects a cell. The rapid and remarkable success of mRNA vaccines in controlling the COVID-19 pandemic has stimulated significant interest in the application of mRNA vaccines. Compared to conventional vaccines using subunit protein antigens or inactivated or attenuated microorganisms, mRNA vaccines have the potential for high potency, expediated development and low-cost manufacture^12,13^. A new coding sequence can be integrated into an mRNA vaccine which can be produced quickly in a scalable cell-free process, making it well-suited for timely control of emerging pandemics. In addition, mRNA does not integrate into the genome, eliminating concerns about insertional mutagenesis^12^.

Lipid nanoparticles (LNPs) are the most commonly used mRNA delivery vehicles^14,15^. Early mRNA vaccine studies using neutral^16^or cationic^17^ liposomes induced T cell responses but resulted in low antibody responses. In contrast, LNP formulations have been shown in numerous studies to elicit both strong T cell responses and robust antibody production^2,18,19^. LNPs provide several advantages for mRNA delivery, such as ease of formulation, biocompatibility, and the ability to carry large or several mRNAs, making it suitable for multivalent mRNA vaccines. Most commonly, mRNA is encapsulated in LNP comprising four major types of lipids: an ionizable lipid, cholesterol, a helper phospholipid, and a PEGylated lipid^20^.

The synthetic cationic lipid DOTMA and its analog DOTAP were the first lipids used for mRNA delivery in 1989^10^. These lipids contain a permanently positively charged quaternary ammonium head group, facilitating the encapsulation of negatively charged nucleic acids. DOTMA and DOTAP have been frequently used for gene therapy and mRNA vaccines^11^, although cytotoxicity in vivo has been reported, which may result from interactions with negatively charged serum proteins^21–24^. Furthermore, the permanently positively charged lipids may activate complement system^25,26^ and are rapidly cleared from the circulation following i.v. administration resulting in a shortened half-life^27^.

To address these challenges posed by the permanently positively charged cationic lipids, numerous efforts have led to the development of ionizable cationic lipids^23,28–32^. These lipids contain one or more pH-responsive ionizable tertiary amines in the head group, acquiring a cationic charge only when the pH is below the acid-base dissociation constant (pK_a_). This transient cationic charge facilitates its release by the low pH of the endosome. These neutral lipids under physiological condition interact less with the anionic membranes of cells, thereby improving the biocompatibility and potentially extending the circulation time of LNPs^11,33^.

In addition to the ionizable lipid, three helper lipid constituents—cholesterol, phospholipids (such as DSPC, DOPC, and DOPE), and PEGylated lipid—also play key roles in facilitating nanoparticle formation and function. Cholesterol improves nanoparticle stability by filling the gaps or voids between lipids and supports fusion with the endosomal membrane during cellular uptake^34^. Helper phospholipids serve as building blocks of lipid bilayer structure, while DOPE enhances efficacy by promoting membrane fusion with the cell and/or endosome^35^. However few studies have compared the effect of phospholipids in combination with ionizable lipids. Finally, the PEGylated lipid consists of polyethylene glycol (PEG) attached to an anchoring lipid, such as DMPE or DMG. The hydrophilic nature of PEG helps stabilize LNPs and regulate nanoparticle size by limiting lipid fusion^36^. Cholesterol, DSPC and PEGylated lipid are components of helper lipids used in the FDA-approved LNP siRNA therapy, patisiran (Onpattro), and have also been included in the FDA-approved SARS-CoV-2 vaccines, mRNA-1273 and BNT162b2. Other synthetic helper lipids have also been identified to enhance vesicle fusion or improve endosomal escape^37,38^, or to help target specific organs such as lungs or spleens^39,40^. However, there still remain several critical knowledge gaps in their application, particularly in optimizing LNP compositions for mRNA delivery, improving stability, and enhancing immune responses in animal models.

This study aims to address these gaps by focusing on the development and optimization of LNP synthesis for an enhanced immune response. Specifically, we evaluated different buffers for improved LNP stability, screened the permanently charged DOTMA and five ionizable lipids with different structural properties and pKa values, and compared DSPC and DOPE along with varying percentages of three lipid components to assess their ability to trigger immune responses. Based on the LNP synthesis process we developed, we constructed a bivalent Fluvid mRNA vaccine and demonstrated the immune response and protective efficacy of this bivalent mRNA vaccine in animal models against influenza and SARS-CoV-2 infections.

## Results

### Buffer evaluation for LNP development

**Fig. 1:**
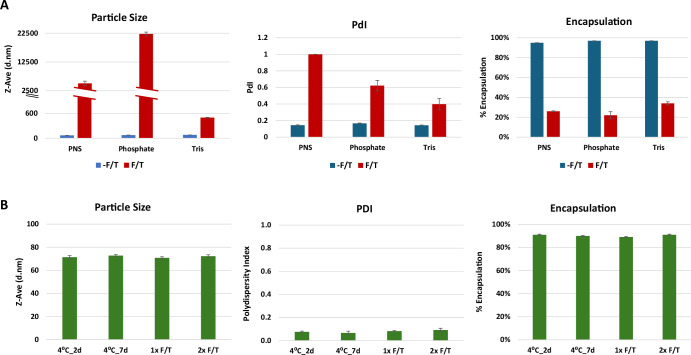
Buffer optimization. **A** Freeze-Thaw (F/T) effect on particle size, polydispersity index (PDI) and encapsulation of GFP mRNA LNPs prepared in different buffers (PNS buffer, phosphate-based buffer and Tris-based buffer). **B** 10% sucrose stabilizes mRNA LNP stored at 4 °C for 2 or 7 days, or upon 1 or 2 Freeze-Thaw (F/T) cycles. mRNA LNP was prepared in Tris/acetate pH 7.4 containing 10% sucrose.

A GFP mRNA LNP containing ALC0315 ionizable lipid was constructed as described in Methods. Three storage buffers were first evaluated for LNP preparations: Phosphate-based buffer (8 mM Phosphate/105 mM NaCl buffer, pH 7.4), Tris-based buffer (20 mM Tris/4.3 mM Acetate buffer, pH 7.4) and PNS buffer (proprietary buffer offered by Precision NanoSystems). All three buffers performed equally well in producing LNPs with similar particle size, polydispersity index (PDI) and encapsulation efficiency (Fig. 1A). However, after a single freeze-thaw cycle, particle size increased, with a higher PDI and significant encapsulation loss, likely due to LNP rupture and aggregation (Fig. 1A). The Tris-based buffer was chosen for further development due to the least changes in particle size and PDI compared to the other two buffers.

We then tested if adding a cryoprotectant in the storage buffer would preserve stability of LNP. A 10% sucrose solution, a pharmaceutical acceptable injectable excipient, was chosen and incorporated into the LNP storage buffer. It was found that the inclusion of 10% sucrose in the LNP storage buffer could effectively maintain the particle size, PDI and encapsulation efficiency stored at 4 °C for 7 days, or after 1 or 2 freeze-thaw cycles (Fig. 1B). Furthermore, transfection efficiency using mRNA LNPs after two freeze-thaw cycles remained comparable to that of freshly prepared mRNA LNPs (Supplementary Fig. 1). The final buffer formulation was therefore determined to be 20 mM Tris/4.3 mM Acetate/10% sucrose (TAS), pH 7.4.

### Comparison of six cationic lipids

**Fig. 2:**
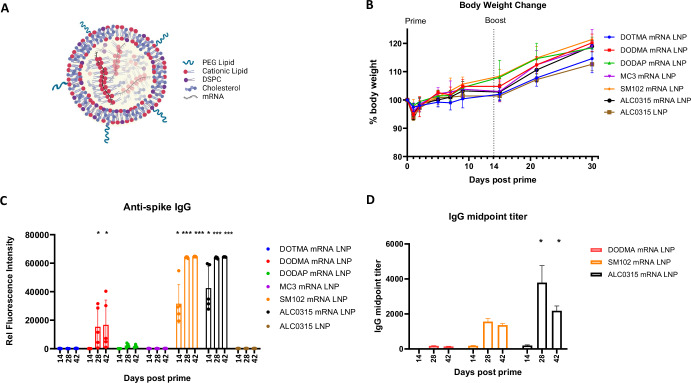
Characterization and comparison of various spike mRNA LNPs made of six different cationic lipids. **A** Schematic of proposed mRNA Lipid nanoparticle structure. All LNPs were produced as described in “Methods”. **B** Weight change after immunizations. Mice (*n* = 5 per group) received two intramuscular injections at d0 (prime) and d14 (boost). **C** Anti-spike IgG at d14, d28, and d42 post prime immunizations. **D** Spike- specific IgG midpoint titers determined by Sigmoidal fit model from plasma titrations on Array. Statistical analysis was performed between DOTMA mRNA LNP group and other groups on each time point. *, *p* < 0.05; **, *p* < 0.01; ***, *p* < 0.001. Only significant *p* values are shown.

LNPs formulated with six different cationic lipids (structures shown in Table 1) were used to encapsulate unmodified spike mRNA encoding the full-length native spike protein from the SARS-CoV-2 Wuhan strain, and were screened for their ability to elicit antibody responses in mice. All LNPs were produced using NanoAssemblr Ignite instrument (Precision Nanosystems) at an N/P ratio = 6 using Cationic Lipid/DSPC/CHOL/PEG lipid (50/10/38.5/1.5 mole%). A schematic of the LNP is shown in Fig. 2A.

**Table 1:** Chemical structures of six cationic lipids and colipids

Cationic lipids	Structure
DOTMA	
DODMA (pKa = 6.59)	
DODAP (pKa = 5.62)	
Dlin-MC3-DMA (pKa = 6.57)	
ALC0315 (pKa = 6.09)	
SM102 (pKa = 6.75)	
**Co-Lipids**	**Structure**
DSPC	
Cholesterol	
DMG-PEG2000	

pKa of cationic lipids was determined by TNS fluorescence titration method.

All LNPs ranged in size from 63 to 149 nm in diameter (Table 2), with the placebo LNPs being the largest, likely due to the absence of mRNA complexation. The PDI was below 0.323, indicating a uniform particle size distribution. As anticipated, the permanent cationic lipid DOTMA LNP exhibited the highest zeta potential at 20.7 mV. Notably, all mRNA LNPs demonstrated an encapsulation efficiency greater than 88%.

**Table 2:** Immunization groups of unmodified spike mRNA LNPs

#	LNP	mRNA	*Z*-avg (d.nm)	Mean PDI	Mean zeta potential (mV)	% Encapsulation
**1**	DOTMA	Spike mRNA	79	0.147	20.7	99%
**2**	DODMA	Spike mRNA	112	0.210	2.6	88%
**3**	DODAP	Spike mRNA	69	0.139	-5.8	97%
**4**	MC3	Spike mRNA	69	0.323	0.4	97%
**5**	SM102	Spike mRNA	75	0.291	0.9	96%
**6**	ALC0315	Spike mRNA	63	0.116	-5.8	98%
**7**	ALC0315	None	149	0.266	-0.2	NA

All LNPs were produced using NanoAssemblr Ignite instrument (Precision Nanosystems) at an N/P ratio = 6 using Cationic Lipid/DSPC/CHOL/PEG lipid (50/10/38.5/1.5 mole%). Unmodified spike mRNA encoding the full-length native spike protein from the SARS-CoV-2 Wuhan strain was used in this study. *N* = 5 per group.

Reactogenicity and immunogenicity were then assessed in female BALB/c mice (4–6 weeks old). Mice (*n* = 5/group) were intramuscularly administered 5 µg of unmodified spike mRNA encapsulated in various LNPs within a 50 µL volume (Supplementary Table 1) on d0 (prime) and d14 (boost). The formulation groups are shown in Table 2.

Reactogenicity was assessed by measuring body weights after prime and boost for 28 days. A slight transient weight loss (3–6%) was observed in all LNP groups after the priming dose. No significant difference was observed between the mRNA-encapsulating ionizable lipid groups and the permanently charged DOTMA mRNA LNP group. However, the empty ALC LNP group exhibited slightly greater weight loss compared to the DOTMA mRNA LNP group (*p* = 0.02). All mice regained weight by the following day (Fig. 2B). No weight loss occurred after the booster dose, indicating that all LNP formulations were well tolerated in mice.

Serological analysis was performed on blood samples taken on days 14, 28, and 42. A protein microarray was used initially to measure spike- specific IgG. As shown in Fig. 2C, ALC0315, SM102, and DODMA LNPs induced the strongest anti-spike IgG responses. By day 14, mice immunized with ALC0315 LNP exhibited the highest anti-spike IgG reactivity, closely followed by those immunized with SM102 LNP. No IgG activity was detected in other cationic lipid LNP groups, such as DOTMA, DODMA, DODAP, and MC3. These findings suggest that the immune responses of mRNA LNPs are highly dependent on the chemical structure of the ionizable cationic lipid. The dendrimeric-like structure (in ALC0315 and SM102) may play a crucial role in enhancing immune response. (Refer to Table 1 for structures). By day 28 (14 days post-boost), anti-spike IgG levels plateaued in the ALC0315 and SM102 LNP groups, with notable IgG reactivity emerging in the DODMA LNP group and showing a slight increase by day 42 (Fig. 2C).

Arrays were then probed to assess IgG titers elicted by ALC0315, SM102, and DODMA LNPs using serial dilutions of the samples. Midpoint titer was determined by Sigmoidal fit model in Fig. 2D. Results confirmed that ALC0315 induced the highest anti-spike IgG, followed by SM102, and then DODMA.

To evaluate the durability of the antibody response, we conducted further probing on microarray on blood collected on day 91 from the top three performing cationic lipid LNP groups: ALC0315, SM102, and DODMA. The anti-spike IgG reactivity remained at a comparable level on days 28 and 42, with only a slight decrease detected on day 91 (Supplementary Fig. 2).

**Fig. 3:**
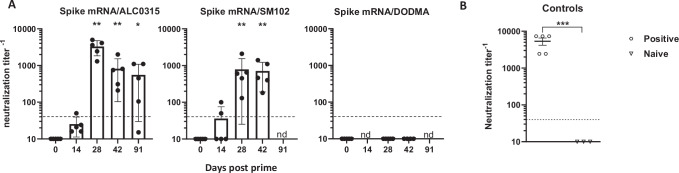
Neutralizing antibodies elicited by the top three LNP cationic lipids. **A** SARS-CoV-2 Virus microneutralization assay on plasma samples collected at different time points from mice immunized with unmodified spike mRNA LNPs containing top ionizable lipids (ALC0315, SM102 or DODMA). Mice (*n* =5 per group) received two intramuscular injections at d0 (prime) and d14 (boost). Dashed line indicates the cutoff titer of 40. **B** Controls. Positive, hyperimmune plasma, *n* = 5. Naïve, normal mouse plasma, *n* = 3. Statistical analysis was performed between formulation groups vs Naïve. *, *p* < 0.05; **, *p* < 0.01; ***, *p* < 0.001. Only significant *p* values are shown. nd, not determined.

We then performed virus microneutralization (MN) assays on blood samples from mice administered spike mRNA encapsulated in LNPs containing ALC-0315, SM-102, or DODMA. The highest neutralizing antibody (nAb) titers (i.e., the highest plasma dilution at which over 50% inhibition is observed) were induced by ALC0315, followed by SM102. By contrast, DODMA mRNA LNP didn’t induce detectable neutralization activity. (Fig. 3). By d91, the neutralization titers from the ALC0315 group decreased slightly compared to d42 (Fig. 3). This result appears to be correlated to anti-spike IgG level observed. As expected, the positive control hyperimmune sample exhibited strong neutralization titer whereas the normal naïve mouse sample showed no virus neutralization ability as expected.

### Optimizing DODMA LNP formulations

DODMA, an ionizable cationic lipid available in the public domain, produced a notably stronger antibody response compared to the other commonly used cationic lipids, DOTMA and DODAP, when used for encapsulating spike mRNA (Fig. 2C). However, this antibody response was not as high as that achieved with the proprietary cationic lipids used in the Pfizer-BioNTech and Moderna vaccines, ALC0315 and SM102, respectively. Therefore, it was of interest to evaluate whether the performance of the DODMA LNP formulations could be enhanced. For this, we assessed DOPE, a commonly used fusogenic phospholipid, to replace DSPC as a co-lipid as DOPE has been shown to promote membrane fusion and release of oligonucleotides from endosome into cytosol^35^. Different percentages of DOPE and cholesterol were also tested. This comparative study of DOPE and DSPC aimed to identify lipid compositions that promote a stronger immune response.

Three DODMA lipid films with 10%, 25%, or 48.5% DOPE were therefore prepared with the following detailed composition: (1) DODMA/DOPE/CHOL/PEG lipid (50/10/38.5/1.5); (2) DODMA/DOPE/CHOL/PEG lipid (50/25/23.5/1.5); and (3) DODMA/DOPE/PEG lipid (50/48.5/1.5).

**Fig. 4:**
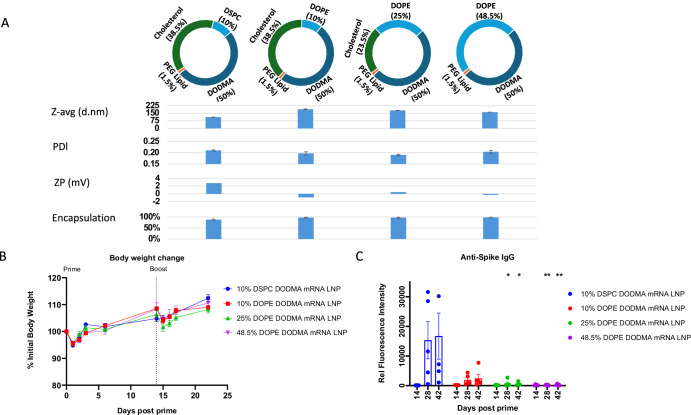
Three DODMA DOPE formulations with various compositions. **A** Particle size, polydispersity index (PDI), zeta potential (ZP) and encapsulation efficiency. All LNPs were produced as described in “Methods”. **B** Body weight change following intramuscular immunizations. **C** Antibody profiling of d14, d28, and d42 plasma samples for anti-spike IgG reactivity. Statistical analysis was performed between 10% DSPC mRNA LNP group and other groups for each corresponding time point. *, *p* < 0.05; **, *p* < 0.01. Only significant p values are shown. *N* = 5 per group.

All LNPs exhibited typical physical characteristics regarding % encapsulation, PDI, and zeta potential, except for the larger particle sizes (ranging from 162 to 190 nm) observed in the DOPE-containing DODMA LNPs (Fig. 4A). We speculate that DOPE has an effect in packing mRNA in the nanoparticle as compared to DSPC which is commonly used in forming LNP.

LNPs were then made with unmodified spike mRNA and the immunological responses were evaluated in C57BL/6 mice. Mice were immunized with 5 µg mRNA LNP on d0 (prime) and d14 (boost) via intramuscular administration. Body weight was monitored for up to 7 days post each immunization. Some weight loss (4 ~ 5%) was observed the day after injection (either prime or boost) but recovered well over the following 1-2 days (Fig. 4B).

The anti-spike IgG response from blood collected at days 14, 28, and 42 was measured using a protein microarray. Unexpectedly, DODMA LNPs with different DOPE compositions did not induce robust anti-spike IgG on day 28 or 42, 14 or 28 days post-boost. The antibody levels were significantly lower compared to the DSPC-containing LNP group (DODMA/DSPC/CHOL/PEG lipid ratio of 50/10/38.5/1.5) (Fig. 4C). This contradicts the hypothesis that DOPE facilitates efficient mRNA release from the endosome for an enhanced immune response. We speculate that the greater lipid bilayer stability provided by DSPC plays a more significant role in enhancing the immune response.

Since altering the composition of the DODMA LNPs did not improve immunogenicity, we returned to the top formulation identified in this study, ALC0315 LNP, with TAS buffer (pH7.4) for mRNA LNP synthesis.

### Chemical modification of mRNA

**Fig. 5:**
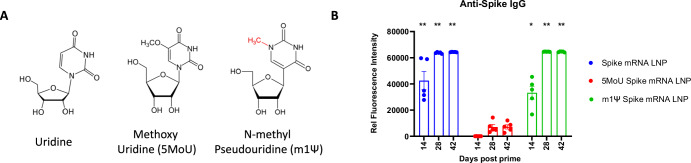
Uridine unmodified vs modified mRNA LNPs. **A** Structures of uridine, methoxy uridine (5MoU) and N1-methyl pseudouridine (m1Ψ). **B** Time course of IgG antibodies induced by mRNA LNPs encapsulated with unmodified or chemically modified spike mRNA. Statistical analysis was performed between 5MoU mRNA LNP group and other groups on the same time points. *, *p* < 0.05; **, *p* < 0.01. Only significant *p* values are shown. *N* = 5 per group.

Next, we assessed immunogenicity resulting from chemical modification of mRNA using ALC0315 LNP. To do this, mRNA was synthesized with unmodified, methoxy (5MoU), and N1-methyl-pseudouridine modified (m1Ψ) uridines as shown in Fig. 5A. Bioanalyzer profiles indicate pure products of mRNA (not shown); similar levels of protein expression were detected from HEK293 cells transfected with these mRNA products (not shown). While no differences were observed in particle size, PDI, or % encapsulation among the three LNPs (not shown), the m1Ψ modification significantly induced anti-spike IgG activity in C57BL/6 mice using ALC0315 LNPs (Fig. 5B). This result aligns with the strong immunogenicity induced in vaccinated populations by the Pfizer-BioNTech and Moderna spike mRNA vaccines, which also use this modification^41^. Interestingly, the unmodified spike mRNA-ALC LNP induced a similar level of anti-spike IgG response on day 14 before the boost, which plateaued on day 28 (14 days post-boost). In contrast, the 5MoU mRNA LNP did not elicit any anti-spike IgG reactivity on day 14 and showed only a slight increase in reactivity on days 28 and 42 (Fig. 5).

### Evaluation of a bivalent Fluvid mRNA vaccine

Based on the mRNA LNP synthesis process we developed, an HA mRNA LNP encoding HA (Influenza A/California/07/2009 (H1N1)) lacking a transmembrane domain, as well as a spike mRNA LNP encoding full-length native spike (SARS-CoV-2 Wuhan strain), were produced using Precision NanoSystems NanoAsemblr. Both mRNAs contained unmodified uridines. The lipids contained ALC0315/DSPC/CHOL/DMG-PEG2000 at 50/10/38.5/1.5 mole. As shown in Supplementary Table 2, the HA mRNA LNP had a particle size of 70 nm, a PDI of 0.255, and an encapsulation efficiency of 97%. Meanwhile, the spike mRNA LNP exhibited a comparable particle size of 74 nm, a PDI of 0.218, and an encapsulation efficiency of 95%.

Seven-week-old BALB/c mice were immunized with monovalent or bivalent (Fluvid) mRNA LNP formulations on d0 and d14 via intramuscular injections (i.m.). For comparison, another group of mice was immunized intramuscularly with HA (H1) and spike (S-2P) protein adjuvanted with IVAX-1, a combination adjuvant with CpG and MPLA in AddaVax nanoemulsion^42^. Plasma samples were collected at multiple time points for assessing immune responses. On day 35, half of the mice (*n* = 5/group) were challenged with H1N1 influenza virus at Animal Biosafety Level 2+ (ABSL2 + ), while the other half were challenged with the SARS-CoV-2 mouse-adapted virus 10 (MA10) on day 42 at ABSL3. The study design is shown in Supplementary Fig. 3 and the formulation groups are shown in Table 3.

**Table 3:** The bivalent Fluvid mRNA LNP study groups

Group	Antigen	Adjuvant	Ag dose/mouse	*N*/group
1	Buffer	-	-	10
2	HA mRNA	ALC0315 LNP	5 μg	10
3	Spike mRNA	ALC0315 LNP	5 μg	10
4	HA + Spike mRNA	ALC0315 LNP	5 μg each	10
5	HA + Spike (S-2P) protein	IVAX-1	5 μg each	10

The HA from A/California/07/2009 (H1N1) strain and spike from SARS-CoV-2 Wuhan strain were used in this study. Both HA and spike mRNAs were unmodified.

To evaluate systemic adverse effects of formulations, mice were monitored for weight changes and signs of morbidity for up to 7 days post immunizations. No distress or changes of behavior were observed, although mice initially experienced weight loss (2–9%) following the prime immunization. The most significant weight loss was observed in the bivalent mRNA LNP and protein/IVAX-1 groups. After boost, only marginal weight loss ( <5%) was observed. All mice regained weight quickly after initial weight loss post immunizations (Supplementary Fig. 3).

**Fig. 6:**
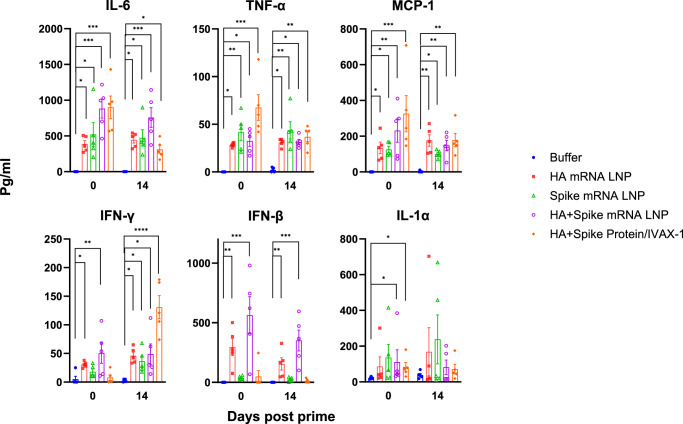
Multiplex cytokine profiling of the blood at 3 h post prime (d0) and 3 h post boost (d14) (*n* = 5). Statistical analysis of various groups vs Buffer is shown on selected cytokines, using Kruskal–Wallis test with Dunn’s multiple groups correction. The full statistical analysis for all 13 cytokines is shown in Table 4. *, *p* < 0.05; **, *p* < 0.01; ***, *p* < 0.001; ****, *p* < 0.0001.

In order to compare the inflammatory cytokines induced by various formulations, a multiplex cytokine assay of the blood at 3 h post prime and 3 h post boost after i.m. injection was performed and revealed a wide range of inflammatory cytokines in all mRNA LNP and protein vaccine groups (Table 4). The bivalent mRNA LNP and protein/IVAX-1 produced the greatest breadth and magnitude of inflammatory cytokines, including IL-6, TNF-α, MCP-1, and IFN-γ, which appears to be correlated with more weight loss observed in these two groups post prime immunization (Supplementary Fig. 3B). The monovalent mRNA vaccine groups induced lower cytokine levels, likely due to the overall half-dose of mRNA in the formulation. Both bivalent mRNA and protein groups produced more IL-1α than the buffer group, and interestingly, the HA mRNA LNP produced more IFN-β than the protein group (Fig. 6). However, there was no significant difference in the production of IL-27, IL-23, IL-10, IL-1β, GM-CSF, IL-17A, or IL-12p70 between the vaccine groups and the buffer control.

**Table 4:** Comparison of cytokines in blood 3 h post immunizations (Vaccine groups vs Buffer)

	vs Buffer													
	Groups	IL-6	MCP-1	TNF-α	IFN-γ	IFN-β	IL-1α	IL-27	IL-23	IL-10	IL-1β	GM-CSF	IL-17A	IL-12p70
**Post Prime**	**HA mRNA LNP**	*	*	*	*	**	ns	ns	ns	ns	ns	ns	ns	ns
	**Spike mRNA LNP**	*	*	**	ns	ns	ns	ns	ns	ns	ns	ns	ns	ns
	**HA+Spike mRNA LNP**	***	**	*	**	***	*	ns	ns	ns	ns	ns	ns	ns
	**HA+Spike Protein/IVAX-1**	***	***	***	ns	ns	*	ns	ns	ns	ns	ns	ns	ns
**Post Boost**	**HA mRNA LNP**	*	**	*	*	**	ns	ns	ns	ns	ns	ns	ns	ns
	**Spike mRNA LNP**	*	*	**	*	ns	ns	ns	ns	ns	ns	ns	ns	ns
	**HA+Spike mRNA LNP**	***	**	*	*	***	ns	ns	ns	ns	ns	ns	ns	ns
	**HA+Spike Protein/IVAX-1**	*	**	**	****	ns	ns	ns	ns	ns	ns	ns	ns	ns

Kruskal–Wallis test with Dunn’s multiple groups correction was performed. ns, not significant; *, *p* < 0.05; **, *p* < 0.01; ***, *p* < 0.001; ****, *p* < 0.0001.

**Fig. 7:**
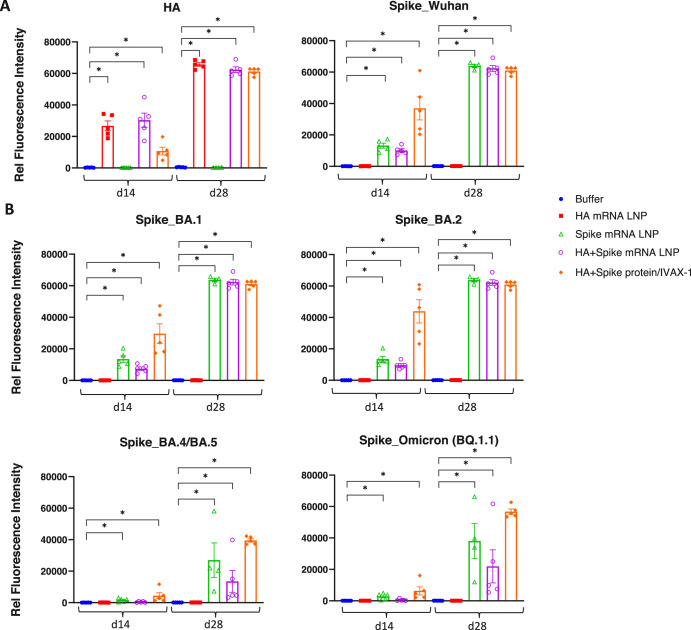
Antibody responses induced by mRNA LNP formulations. **A** Total IgG responses against HA (H1N1) and spike (Wuhan strain) were detected from blood samples collected at d14 and d28 on protein microarray. **B** Spike (variant)- specific IgG antibody response. Statistical analysis of various groups vs Buffer was performed using Kruskal–Wallis test with Dunn’s multiple groups correction. *, *p* < 0.05; **, *p* < 0.01; ***, *p* < 0.001; ****, *p* < 0.0001. *N* = 5 per group.

Blood samples were collected on d14 and d28 (Fig. 7A). A strong IgG response against the HA antigen was observed on day 14 (single dose) in mice immunized with either HA mRNA LNP or IVAX-1adjuvanted HA protein. The HA mRNA groups (both monovalent and bivalent) generated a higher antibody response than the protein group after a single dose. Following a boost on day 14, the IgG response plateaued by day 28. As expected, no HA-specific antibodies were produced in mice that received the monovalent spike mRNA LNP (Fig. 7A).

IgG against the immunizing spike antigen (Wuhan strain) was detected on day 14 in mice immunized with IVAX-1-adjuvanted spike protein (S-2P) or spike mRNA LNP. An elevated response was observed by day 28 in both the spike mRNA LNP groups (monovalent and bivalent) and the adjuvanted protein group (Fig. 7A).

Cross-reactive IgG against spike from variant strains (BA.1, BA.2, BA.4/BA.5, and BQ.1.1) was observed on day 14 in mice immunized with either spike mRNA LNP or IVAX-1-adjuvanted spike protein. The IgG response increased on d28 following a boost on day 14 (Fig. 7B). Our results indicate that combining spike and HA has induced similar levels of antibody response compared to individual antigens.

**Fig. 8:**
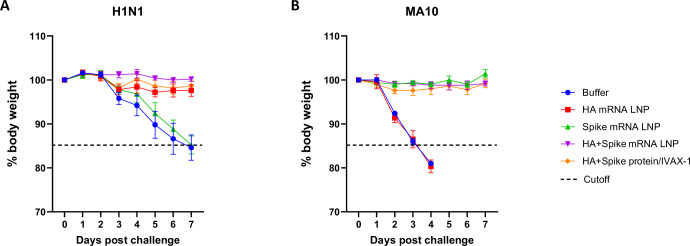
Weight change following viral challenges. **A** Intranasal challenge with influenza H1N1 virus (A/California/07/2009 (H1N1) x A/Puerto Rico/8/1934) at 10^3^ TCID_50_/mL dose, on day 35 post prime immunization. **B** Intranasal challenge with 10^4^ TCID_50_/mL SARS-CoV-2, mouse-adapted, MA10 Variant, on d42 post prime immunization. *N* = 5 per group.

Protective efficacy was evaluated after a challenge with H1N1 virus at d35 or SARS-CoV-2 (MA10) virus at d42. Female BALB/c mice from each group (*n* = 5/group) were challenged with virus (A/California/07/2009 (H1N1) x A/Puerto Rico/8/1934) on d35. The dose-ranging study revealed that a dose of 10^3^ tissue culture infectious dose 50 (TCID_50_)/mL was lethal to unvaccinated BALB/c mice, whereas a dose of 10^2^ TCID_50_/mL was lethal to four out of five unvaccinated BALB/c mice (Supplementary Fig. 4). Therefore, a dose of 10^3^ TCID_50_/mL of H1N1 virus was selected for use in BALB/c mice for our animal challenge study. These mice receiving HA mRNA LNP or HA protein formulated with IVAX-1 produced a robust IgG response (Fig. 7). After H1N1 viral challenge, body weight of mice was monitored for 7 days. As shown in Fig. 8 A, as expected, all mice receiving adjuvanted HA protein or HA mRNA LNP (monovalent or bivalent) were protected from H1N1 challenge without losing weight for up to 7 days post challenge. Conversely, weight loss was observed in PBS and spike mRNA LNP control groups. No significant difference in weight change (*p* = 0.21) or morbidity was observed between mice receiving the monovalent and bivalent HA mRNA LNPs.

On d42 following prime immunization, the remaining mice from each group (*n* = 5/group) were intranasally challenged with a lethal dose (10^4^ TCID_50_/mL, as determined by dose ranging study in Supplementary Fig. 4B) of mouse adapted SARS-CoV-2 virus (MA10) at ABSL3. The body weight of the mice was monitored for 7 days. Mice that received the full-length spike mRNA LNP formulations (monovalent or bivalent) or S-2P protein formulated with IVAX-1 generated a strong IgG response (Fig. 7). All mice in these three groups were protected from SARS-CoV-2 challenge, maintaining their weight for up to 7 days post-challenge. In contrast, mice in the PBS and H1 mRNA LNP control groups lost more than 15% of their weight. (Fig. 8B). Likewise, there was no significant difference in the weight change (*p* = 0.13) for up to 7 days post challenge or morbidity for mice receiving the monovalent or bivalent spike mRNA LNPs.

### T cell recall response elicited by mRNA LNP formulations

**Fig. 9:**
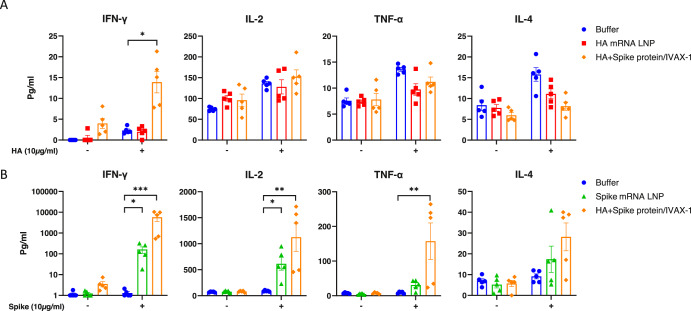
Multiplex cytokine profiling in overnight stimulated splenocyte cultures at 7 days post boost immunization. Mouse splenocytes were stimulated with 10μg/mL (**A**) HA(H1N1) or (**B**) spike (Wuhan strain) for 18 h. Comparison between immunized groups vs PBS control group was performed using a Kruskal–Wallis test with Dunn’s multiple groups correction. The statistical analysis for all 12 cytokines is shown in Table 5. *, *p* < 0.05; **, *p* < 0.01; ***, *p* < 0.001; ****, *p* < 0.0001. All other comparisons were non-significant. *N* = 5 per group.

To investigate the T cell response induced by mRNA LNP formulations, mice were administered monovalent HA or spike mRNA encapsulated in ALC0315 LNPs via intramuscular injections on d0 and d14, and spleens were harvested on d21 (7 days post boost immunization). An HA/spike protein formulation adjuvanted with IVAX-1 was included as a control. Splenocytes were stimulated overnight with 10μg/mL HA or spike proteins respectively. After 18 h, the cell cultures were harvested and a LEGENDplex assay was performed to measure the cytokines in the cultures. As shown in Fig. 9 and Table 5, the IVAX-1- adjuvanted HA group induced IFN-γ production but no other cytokines, indicating a Th1 biased response, however the HA mRNA LNP formulation didn’t induce significantly higher cytokine production upon HA antigen recall. On the other hand, the spike antigen stimulated a much higher recall response compared to the HA antigen. IFN-γ, IL-2, TNF-α were significantly higher in spike mRNA LNP and IVAX-1- adjuvanted spike protein groups compared to buffer control group, and no IL-4 was elicited in any groups, suggesting that a Th1 biased response is achieved with both spike mRNA LNP and IVAX-1- adjuvanted spike protein formulations. Meanwhile, IL-6 secretion was also elevated in the vaccine formulation groups upon recall with the spike antigen, suggesting effective promotion of T cell differentiation (Table 5).

**Table 5:** Cytokines in the overnight stimulated splenocyte cultures

	vs Buffer												
	Groups	IFN-γ	TNF-α	IL-2	IL-4	IL-5	IL-6	IL-10	IL-9 (B3)	IL-17A	IL-17F	IL-22	IL-13
HA	HA mRNA LNP	ns	ns	ns	ns	ns	ns	ns	ns	ns	ns	ns	ns
	HA+Spike protein/IVAX-1	*	ns	ns	ns	ns	ns	ns	ns	ns	ns	ns	ns
Spike	Spike mRNA LNP	*	ns	*	ns	*	*	ns	ns	ns	ns	ns	ns
	HA+Spike protein/IVAX-1	***	**	**	ns	ns	**	ns	ns	ns	ns	ns	ns

Splenocytes were isolated from mice receiving different formulations and stimulated with 10 μg/mL HA or 10 μg/mL spike for 18 h. The Statistical analysis of various groups vs Buffer is performed using Kruskal–Wallis test with Dunn’s multiple groups correction. *, *p* < 0.05; **, *p* < 0.01; ***, *p* < 0.001; ****, *p* < 0.0001.

## Discussion

In this study, we have compared six cationic lipids and helper lipids for their ability to elicit immune response from mRNA. No significant weight loss or systemic adverse effects were observed in mice for any of the lipids tested. In contrast, the immunogenicity of the lipids in vivo varied widely. Thus, ionizable lipids ALC0315, SM102, and DODMA triggered an antibody response, whereas ionizable lipids MC3, DODAP, and permanently cationic DOTMA did not. When co-encapsulated with DODMA, the phospholipid DSPC demonstrated enhanced immunogenicity compared to DOPE. These data align with other studies showing SM102 as more effective than MC3 for mRNA delivery^43^, indicating that LNP formulations designed for siRNA delivery may not be optimal for mRNA and DNA delivery^44,45^. Additionally, other research reported no detectable protein expression or immunogenicity for LNPs containing DOTAP, an analog of DOTMA^45^.

The varied performance of the different cationic lipids in vivo might be attributed to several factors. First, ionizable cationic lipids at physiological pH, similar to neutral lipids, may interact less with the blood proteins or anionic membranes of cells compared to permanently positively charged lipids, such as DOTMA^23,28–32^. This may enhance the biocompatibility of neutrally-charge lipids and increase half-life in vivo^15,31,32^. However, in the acidic endosome, ionization provides amphiphilicity to the lipids, which destabilizes the endosomal membrane, facilitating endosomal escape^46^. Second, the structure of lipids such as the numbers of branches and the branching position of the aliphatic chain with respect to the polar head group plays an important role in efficient delivery in vivo^47^. ALC0315 and SM102 contain three to four branched lipid tails compared to other cationic lipids, such as MC3^15^, which may affect lipid phase transition and thus enhance endosomal escape properties. Studies examining the roles of DOPE and DSPC in LNP formulations often note that while DOPE has a smaller head group and is fusogenic, DSPC tends to contribute to more stable and efficient LNP delivery due to its larger head group and less fusogenic nature. In contrast, DOPE, with its smaller head group and fusogenic properties, is better for promoting endosomal escape but can sometimes reduce stability in vivo^48,49^. Meanwhile, the p*K*_a_ of the ionizable lipid is critical for cellular uptake, and endosomal escape efficiency. The optimal p*K*_a_ for mRNA delivery to and expression in the liver has been reported to be between 6.0 and 6.8^44,45^ whereas DODAP has a lower pKA at 5.62, which might reduce the endosomal escape efficiency.

Several studies have used nucleoside modified mRNA, such as pseudouridine, N1-methylpseudouridine (m1Ψ) or other nucleoside analogs^50^, to avoid recognition by pattern recognition receptors, such as Toll-like receptor 3 (TLR3), TLR7 and TLR8, and the retinoic acid-inducible gene I (RIG-I) receptor^51^. Nucleoside-modified mRNAs have been shown to enable efficient protein translation^12,18,52^ and induce potent T follicular helper cells and germinal center responses^18^. Both the Moderna and Pfizer–BioNTech SARS-CoV-2 vaccines, which produced >94% efficacy in phase III clinical trials^53^, contain N1-methylpseudouridine mRNAs.

On the contrary, Kauffman et al. demonstrated that pseudouridine substitution of mRNA did not change the in vivo protein expression, or mRNA immunogenicity as compared to unmodified mRNA when administered intravenously via liver targeting LNPs^54^. Thess et al. reported that sequence engineered mRNA without chemical modification of nucleoside showed higher protein production with mild cytokine induction when compared to pseudouridine-substituted mRNA in mice^55^. In a murine melanoma model, unmodified mRNA induced substantial IFN-I production, notably inhibited tumor growth and prolonged survival, compared to m1Ψ modification. This robust anti-tumor effect correlated with the increase in intratumoral CD40 + DCs and the frequency of granzyme B^+^/IFN-γ^+^/TNF-α^+^ polyfunctional antigen-specific CD8 + T cells^56^. Together, these data indicate the effects of modified nucleotides seem inconsistent and may warrant further investigation.

In the present study, we have shown both unmodified and m1Ψ uridine mRNA elicited equivalent level of antibody response, whereas methoxy uridine modified (5MoU) mRNA failed to induce antigen specific antibody response. Although we have not conducted a direct comparison of the inflammatory responses induced by unmodified versus modified mRNA, mild inflammatory cytokine production from the unmodified mRNA LNP was detected compared to the adjuvanted protein formulation. The unmodified mRNA was able to induce Th1 polarized T cell response upon antigen recall and confer 100% protection in mice challenged with influenza or SARS-CoV-2 viruses. Lacking an antibody response production from 5MoU mRNA may not be due to the lower expression of proteins (unpublished data) and may have utility for to gene therapy or gene editing where immunogenicity is not desired. Further studies are needed to address the knowledge gaps regarding nucleoside modifications.

Another intriguing finding from our study is that while the S-2P substitution, which stabilizes protein conformation^57^, is used in several COVID-19 mRNA vaccines^58^, the mRNA encoding the native spike protein also induced strong neutralizing antibodies and provided full protection against SARS-CoV-2 challenge without the 2 P substitutions. Similarly, Prompetchara et al. demonstrated a SARS-CoV-2 mRNA encoding prefusion-unstabilized ectodomain spike protein (Wuhan strain) encapsulated in LNP elicited robust nAb and T cell responses in female BALB/c mice. This formulation also protected human-ACE-2-expressing female mice from SARS-CoV-2 challenge and significantly reduced viral load^59^. Furthermore, a recent study by Malewana et al. reported that nucleoside-modified mRNA encoding the native SARS-CoV-2 spike protein (Wuhan strain), lacking proline substitutions, was capable of broadly neutralizing both macaque and human Omicron variants of concern^60^. These findings are consistent with and support our results. Further studies directly comparing the 2P-stabilized spike protein and mRNA with the native protein and mRNA will help clarify their relative significance.

The weaker T cell response against HA (H1N1) antigen recall may be associated with the poor immunogenicity of the HA (H1N1) antigen^61^ or the secreted antigen encoded by the mRNA in the formulation. It has been reported that T cell responses may vary between mRNA vaccines encoding membrane-bound proteins and those encoding secreted proteins^19,62^. Although both mRNA vaccines encoding secreted and membrane bound spike proteins induced helper CD4 + T cells, CD8 + T cells were only reported for the Pfizer-BioNTech membrane bound mRNA vaccine^19,62^. The significance of this result remains to be determined, as both vaccines elicited comparable levels of protection ( ~ 95%) against SARS-CoV-2 strains circulating at the time clinical trials were performed. The higher IFN-γ secretion observed upon antigen recall in the IVAX-1- adjuvanted protein group (CpG and MPLA in AddaVax nanoemulsion) compared to the mRNA group may be attributed to the adjuvant, as CpG and MPLA are potent TLR9 and TLR4 agonists that strongly activate T cell responses. Meanwhile, in this study, the whole antigen was used for T cell antigen recall, which is likely to be suboptimal for restimulation of CD8 T cells, possibly resulting in an underestimation of true CD8 T cell response^63^. This may be overcome by using antigenic peptides appropriate for CD8 T cells for recall.

Our study also demonstrates that a bivalent Fluvid mRNA vaccine was successfully constructed using the defined procedure for mRNA LNP synthesis in our lab. The bivalent Fluvid mRNA vaccine demonstrated capability of eliciting immune response as well as conferring protective efficacy against both influenza and SARS-CoV-2 virus challenges, without compromising efficacy when compared to respective monovalent vaccines. This underscores the effectiveness of the multivalent mRNA vaccine strategy in addressing multiple diseases within a single formulation.

In summary, our study offers valuable insights into the role of lipid formulations and nucleotide modifications in mRNA delivery and immune response induction. However, several critical aspects remain to be fully understood, particularly the inflammatory responses triggered by unmodified versus modified mRNA, and the role of signal peptides in modulating immune responses for mRNA vaccines. Ongoing research will further investigate how these factors impact mRNA stability, immune responses, and overall vaccine efficacy. Addressing these knowledge gaps will be essential for optimizing mRNA-based vaccines and expanding their clinical applications.

## Materials and methods

### Proteins

Hemagglutinin protein from influenza A H1N1 (A/California/04/2009) was obtained from Sino Biological, Inc. (Wayne, PA; catalog# 11055-V08H). Pre-fusion stabilized spike antigen (S-2P) was obtained from BEI resources (Manassas, VA; catalog# NR-53937). S-2P contains 1194 residues (ectodomain) of the SARS-CoV-2 S glycoprotein; the recombinant protein was stabilized by substitution at the furin S1/S2 cleavage site (RRAR to GSAS; residues 682 to 685) and KV to PP mutations (residues 986 and 987) and includes a T4 foldon trimerization domain^64^.

### mRNA

Unmodified, 5-methoxy uridine modified and 5-methyl pseudouridine modified mRNA encoding the native full-length SARS-CoV-2 spike (S) protein from Wuhan strain (catalog# MRNA34, MRNA35, and MRNA43 respectively), were purchased from OZ Biosciences (San Diego, CA). Unmodified influenza hemagglutinin mRNA was also purchased from OZ Biosciences (catalog# MRNA46) encoding HA from H1N1 (A/California/04/2009) with a deletion in the transmembrane domain. The unmodified GFP mRNA (catalog# 3870-1000) was obtained from Aldevron (Madison, Wisconsin). These mRNAs contain Cap 1 structures along with their proprietary UTR sequences respective to the manufactures.

### Lipids and excipients

Cationic lipids DOTMA (1,2-di-O-octadecenyl-3-trimethylammonium propane), DODMA (1,2-dioleyloxy-3-dimethylaminopropane), DODAP (1,2-dioleoyl-3-dimethylammonium-propane) and ALC-0315 (6-((2-hexyldecanoyl)oxy)-N-(6-((2-hexyldecanoyl)oxy)hexyl)-N-(4-hydroxybutyl)hexan-1-aminium) and co-lipids DSPC (1,2-distearoyl-sn-glycero-3-phosphocholine), DOPE (1,2-dioleoyl-sn-glycero-3-phosphoethanolamine), DMG-PEG 2000 (1,2-dimyristoyl-rac-glycero-3-methoxypolyethylene glycol-2000) and CHOL (cholesterol) were obtained from Avanti Polar Lipids, Inc. (Alabaster, AL, USA). Cationic lipids SM-102 and MC3(DLin-MC3-DMA) were purchased from BroadPharm (San Diego, CA; catalog# BP-25499 and BP-25497, respectively).

Excipients, including Tris base, Tris HCl, sodium acetate, glacial acetic acid, sucrose, monobasic dihydrogen potassium phosphate, and dibasic hydrogen sodium phosphate were purchased from Sigma Aldrich (St. Louis, Missouri). PNS buffer, a proprietary buffer, was obtained from Precision NanoSystems (Vancouver, Canada).

NanoAssemblr NxGen cartridges were purchased from precision nanosystems. Pur-A-Lyzer maxi dialysis kit (catalog# PURX12015) used for LNP dialysis was purchased from Sigma Aldrich. Syringes used for LNP manufacturing were obtained from BD Biosciences (La Jolla, CA).

### Viruses

Influenza A H1N1 Virus (A/California/07/2009 (H1N1) x A/Puerto Rico/8/1934) was obtained from BEI Resources, catalog# NR-44004, and propagated in research grade serum pathogen free chicken eggs (AVS Bio, Norwich, CT) according to previously published protocols^65^. Briefly, a 1/100 dilution of clarified lysate was inoculated into the allantoic cavity of the egg and incubated at 37 °C and 60% humidity. After 48 h, eggs were placed at 4 °C overnight and then allantoic fluid was harvested and centrifuged at 1000x RPM for 10 min. Clarified supernatant was then stored in aliquots at −80 °C.

SARS-Related Coronavirus 2 (SARS-CoV-2), Isolate USA-CA3/2020 and SARS-Related Coronavirus 2, mouse-adapted, MA10 variant (USA-WA1/2020 backbone) were obtained from BEI Resources (catalog #NR-52385 and NR-55329, respectively). Each was supplied as spin- clarified cell lysate and supernatant from infected cells. SARS-CoV-2 USA-CA3/2020 was propagated in VeroE6 cells obtained from ATCC (Manassas, VA; catalog #CRL 1586) according to product information sheet. SARS MA10 variant was propagated in Calu-3 cells (ATCC HTB-55) according to product information sheet. Briefly, cells seeded into 150 cm^2^ flasks were incubated for 16–24 h at 37 °C/5% CO_2_ in a humidified atmosphere. For inoculation, actively growing Vero E6 cells (80–90% confluent) were washed twice with PBS and a 1/50 dilution of inoculation culture was added for 1 h with periodic agitation. Inoculation cultures were prepared from stocks diluted to 1:50 in 2 mL of virus growth media comprising of Eagle’s Minimum Essential Medium (EMEM; ATCC catalog #30-2003), 1% penicillin-streptomycin (GIBCO), and 2% Fetal Calf Serum (ATCC). After washing in PBS, infected cells were cultured for 48–72 h. When >90% of cells showed cytopathic effect (CPE), culture supernatants were harvested and clarified by centrifugation (3200 × g at 4 °C for 10 min), and 0.5–1 mL aliquots stored in cryovials at −80 °C.

### Viral titer

To determine the 50% TCID, established protocols were used as published^66^. Briefly, Vero E6 cells (for SARS-CoV-2) or MDCK cells (ATCC CCL-34) for influenza were sub-cultured on a 96-well plate to 90–100% confluency in virus growth media (EMEM (ATCC) containing 1% Penicillin-Streptomycin (GIBCO), and 2% heat-inactivated fetal calf serum (ATCC). Ten-fold dilutions of the virus are added to the plate and subsequently added to sub-cultured cells. After 1 h of incubation, virus was removed, virus growth media was added to the cells and incubated for 3 days at 37 °C and 5% CO_2_. Three days post infection, cells positive for CPE were noted and the TCID_50_/mL was calculated using the Reed & Muench method^66^.

### mRNA/LNP formulation and characterization

LNPs used in this study contain a cationic lipid/DSPC/cholesterol/DMG-PEG2000 (50:10:38.5:1.5 mol/mol), encapsulated RNA-to-total lipid has an approximate weight ratio of 0.05 and a diameter of 70 ~ 100 nm. MRNA LNP made of permanently positively charged cationic lipid was prepared at neutral pH, while mRNA LNP made of ionizable lipid was prepared at pH4. Briefly, all lipids were dissolved in ethanol and rapidly combined with mRNA in 100 mM sodium acetate (NaOAc) buffer, at pH 4 (ionizable lipid) or neutral pH (permanently positively charged lipid), at a volume ratio of 1:3 (ethanol:aqueous) and charge ratio of cationic lipid to the negatively charged mRNA (N/P ratio) of 6. The combination was performed by microfluidic mixing using NanoAssemblr Ignite device (Precision Nanosystems).

The mRNA LNP formed was then dialyzed using Pur-A-Lyzer maxi dialysis kit with >100 volumes of 20 mM Tris/4.3 mM Acetate/10% Sucrose buffer pH 7.4 (TAS buffer) for at least 5 h to remove ethanol and raise pH to 7.4 to form final mRNA LNP preparation. Other monovalent low ionic strength buffers containing cryoprotectant may also be used. Finished mRNA LNP may be concentrated using centrifugal ultrafilter (if needed), and sterile filtered. All mRNA-LNP formulations were stored at −80 °C at a concentration of mRNA of ~1 μg/μL.

The produced LNPs were characterized for particle size and PDI by dynamic light scattering using Zetasizer ultra from Malvern Panalytical (Malvern, England), mRNA encapsulation by RiboGreen assay in the absence and presence of 2% TritonX100, and mRNA integrity by agarose gel electrophoresis and 2100 Agilent Bioanalyzer (Agilent Technologies, La Jolla, CA).

### mRNA transfection

HEK293-T cells (ATCC, Manassas, VA) were cultured in Dulbecco’s Modified Eagle Medium with Glutamax (DMEM) (Gibco, Waltham, MA) containing 10% fetal bovine serum (FBS) (ATCC) and 1% penicillin-streptomycin (Gibco). For transfections with mRNA samples, cells were seeded at ~100,000 cells/well in a 24 well tissue culture plate (Corning Inc., Corning, NY) 16-24 hr prior to the transfection. Cells were treated with 200 µL of Opti-MEM (Gibco) containing 1 µg mRNA encapsulated in ALC-0315 LNP. The transfected cells were then incubated at 37 °C and 5% CO_2_ in a humidified incubator for 2 h. Twenty percent FBS in Opti-MEM medium was added to each well at 2 h and the plates continued incubation at 37 °C and 5% CO_2_ in a humidified incubator for an additional 22 h. At 24 h, the medium and mRNA treatments were aspirated, cells were washed with PBS (Corning), and then trypsin-EDTA (ATCC) was added and sequentially quenched in complete DMEM medium to collect cells. The cells were then stained with either 7-AAD (fresh LNP) or Ghost Dye UV 450 (Cytek) (frozen LNP) for viability, Ghost Dye treated cells received an additional quenching step with complete medium. All groups received two PBS washes before preparing samples using a Flow Cytometry Staining Kit and a matching protocol (BioLegend, San Diego, CA). Cells were analyzed by spectral flow cytometry using a Cytek Aurora 5-laser flow cytometer (Cytek Biosciences, San Diego, CA). The analysis was done using FlowJo software (BD Biosciences).

### Mouse immunizations and challenges

All animal work was approved by the UCI Institutional Animal Care and Use Committee (IACUC protocol No. AUP-22-032 and AUP-22-028). The laboratory animal resources at UCI are Internationally accredited by the Association for Assessment and Accreditation of Laboratory Animal Care (AAALAC) (AAALAC No. 000238). All experiments were performed in accordance with the animal use protocol approved by IACUC. Female C57BL/6 and BALB/c mice (4–6weeks of age) were purchased from Charles River Inc., and housed in standard cages with enrichment. Formulations were administered at 50 µL dose (5 µg mRNA or protein) via the intramuscular (i.m.) route (caudal thigh) under anesthesia with inhaled isofluorane/O_2_ mixture. The animal was then allowed to regain consciousness in the cabinet and then returned to its cage. Mice were weighed and monitored daily for the first two weeks post immunization for any changes in behavior or appearance and periodically thereafter. Blood was collected into heparinized microcapillary tubes at regular time points (d0, d14, d28, and d42) by facial vein bleed under anesthesia with inhaled isofluorane/O_2_ then centrifuged to obtain plasma in the supernatant. Plasma aliquots were stored at −80 °C until ready for analysis.

### Influenza and SARC-CoV-2 challenge

For intranasal (i.n.) challenge, A/California/07/2009 (H1N1) × A/Puerto Rico/8/1934 or SARS-CoV-2 mouse-adapted MA10 variant virus (USA-WA/1/2020 backbone) was administered at 10^4^ TCID_50_/mL in a total volume of 50 μL using a micropipette pipette to the right-side nares of female BALB/c (Charles River Laboratories) mice 6–8 weeks of age that were transiently anesthetized in an isofluorane/O_2_ mixture, in an ABSL2+ or ABSL3 facility, respectively. Following the challenge procedure, mice were observed until recovery from anesthesia. Daily body weights were taken following H1N1 or SARS-CoV-2 challenge until the end-point (either day 8 post-challenge or earlier if an individual animal lost >15% body weight or showed significant distress) when the animal was euthanized. Euthanasia was performed via CO_2_ inhalation (primary method) followed by secondary physical method (cervical dislocation).

### Serology

#### Protein microarray

A protein microarray containing purified hemagglutinin from H1N1(A/California/07/2009) and spike protein from the original Wuhan and other strains was printed and probed as described^67–69^. Briefly, microarray slides were incubated with mouse plasma samples diluted to 1:100 in blocking buffer (GVS Life Sciences, Sanford, ME), washed and followed with incubation with AF647 conjugated anti- mouse secondary antibody (Jackson ImmunoResearch) diluted 1/200 in blocking buffer. The slides were washed and air-dried by brief centrifugation. Microarray slides were scanned and analyzed using a TinyCAM scanner. Intensities were quantified using ScanArray software. All signal intensities were corrected for spot-specific background.

#### MN assays

MN assays in this study were performed as described^70^. For positive control (hyperimmune) plasma, female C57BL/6 mice were administered 50 µL Pfizer-BioNtech COVID-19 mRNA vaccine remnant via i.m. (caudal thigh) route on d0, d14, and d105 and plasma collected 21 days after the final boost. Briefly, Vero E6 cells (ATCC CRL-1586) were maintained in Eagle’s minimum essential medium (EMEM; ATCC) containing penicillin/streptomycin (Gibco) and 10% heat-inactivated fetal calf serum (ATCC) and cultured at 37 °C with 5% CO_2_ in a humid environment. Cells were sub-cultured when 80–85% confluency. One day prior to assay, Vero E6 cells were sub-cultured into flat-bottomed 96 well plates at 1.0 × 10^4^ cells/well in 100 μL. Mouse plasma was diluted 1/10 in virus growth media (EMEM containing 1% Penicillin-Streptomycin (GIBCO), and 2% heat-inactivated fetal calf serum) then serially diluted (three-fold) in virus-growth medium in a separate 96-well plate. SARS-Related Coronavirus 2, Isolate USA-CA3/2020 virus (BEI) was diluted to 10^4^ TCID_50_/mL in virus growth media and then added to serially diluted supernatants and incubated for 1 h at 37 °C, 5% CO_2_. Wells containing only viruses and growth media were also prepared to serve as controls. Following incubation, media from cell monolayers were replaced with the plasma-virus mixtures and were incubated for an additional 1 h. Plasma-virus mixtures were then replaced with 200 μL of virus growth media and plates were incubated for 48 h at 37 °C. Cells were then fixed in 4% paraformaldehyde in PBS for 30 min, washed in PBS, and then permeabilized in 0.1% PBS/Triton X-100 at RT for 15 min. Cells were washed and blocked in a blocking buffer of 3% BSA (Sigma-Aldrich) in PBS for 1 h at RT. SARS-CoV-2 nucleoprotein (NP) was detected using anti-SARS NP mAb (SinoBiological, 40143) Diluted 1/5000 in blocking buffer, followed by horseradish peroxidase-conjugated anti-mouse IgG (KPL) diluted to 1/5000 in blocking buffer. Plates were developed in 3,3′,5, 5′-tetramethylbenzidine peroxidase substrate (SureBlue) and reactions were quenched using 0.18 M H_2_SO_4_. Assays were quantified in an ELISA plate reader at 450 nm using SoftMax Pro 7.1 software. Titer was determined to be the highest dilution with at least 50% inhibition observed. IC_50_ values were determined using Graphpad Prism 10 (GraphPad, La Jolla, CA, USA) using non-linear regression XY analyses.

### Multiplex cytokine profiling in blood after immunizations

Three hours post prime and boost immunizations, plasma samples were collected from mice and assayed for 13 inflammatory cytokines using LEGENDplex mouse inflammation panel kit from Biolegend (catalog#740446) on a 5-channel Cytek Aurora Spectral Flow Cytometer (Cytek Biosciences), according to the manufacturer’s instructions. The analysis was done using LEGENDplex data analysis software Qognit (Biolegend).

### T cell recall assay

Seven days post boost immunizations, splenocytes from immunized BALB/c mice were harvested. Recall assays were performed as described previously^71^. Antigens used for recall were HA (H1 Cal09) expressed in HEK293 cells from Sino Biological (catalog# 11055-V08H) as well as SARS-CoV-2 spike expressed in CHO cells (BEI resources; catalog#NR-53937) at a concentration of 10 µg/mL. Assays were performed in T cell medium comprising Iscove’s Modified Dulbecco’s Medium, containing 5 × 10^−5 ^M β-mercaptoethanol, 100 IU/mL penicillin, 100 μg/mL streptomycin, and 10% fetal calf serum as described^71^. After 18 h of incubation, the assay supernatants were collected for multiplex cytokine screening using the LEGENDplex Mouse *T* Helper Cytokine Panel (Biolegend; catalog#741044) and analyzed using Qognit software according to the manufacturer’s instructions.

### Statistical analysis

For protein microarray data, quantile normalization was conducted to reduce assay to assay variation as previously described^69^. All data analysis was performed, and figures were generated in the R programing environment (Version 4.4.1, https://www.r-project.org/) or Graphpad Prism 10 (GraphPad, La Jolla, CA, USA). Figure 2A was generated using BioRender software (https://BioRender.com/znlrzos). Statistical analysis was performed using two-tailed Mann-Whitney test, followed with Benjamini and Hochberg correction^72^ for multiple comparisons, or two-tailed Kruskal–Wallis tests with Dunn’s multiple-comparisons. A *P* value of <0.05 was considered statistically significant.

## Supplementary information

Supplementary information

## Data Availability

Microarray data of this study has been deposited into the Gene Expression Omnibus (GEO) (https://www.ncbi.nlm.nih.gov/geo/) under the accession number GSE295393. Other data are provided within the manuscript or supplementary information files.
