# Supplementary material for: Lipid Nanoparticle Development for A Fluvid mRNA Vaccine Targeting Seasonal Influenza and SARS-CoV-2
Source: NPJ Vaccines. 2025 Jun 11;10:123. doi: 10.1038/s41541-025-01153-6 (PMC12159160; doi:10.1038/s41541-025-01153-6)
Supplement: Supplementary file 1 — Supplementary information [file 41541_2025_1153_MOESM1_ESM.pdf]

Supplementary Tables and Figures

	mRNA dose (µg)	Total lipids (µg)	Lipid/mRNA (wt ratio)
DOTMA LNP	5	109	21.8
DODMA LNP	5	105	20.9
DODAP LNP	5	107	21.4
MC3 LNP	5	107	21.3
ALC0315 LNP	5	118	23.6
SM102 LNP	5	113	22.6

Supplementary Table 1. Summary of total lipids in 50µL injection volume with 5 µg mRNA encapsulated and used for immunization. Encapsulation was done at an N/P ratio=6.

Sample Name	Avg d.nm	Avg PdI	Avg Encapsulation
HA mRNA LNP	70	0.255	97%
Spike mRNA LNP	74	0.218	95%

Supplementary Table 2. Characterization of HA and spike mRNA LNPs. N=5 per group.

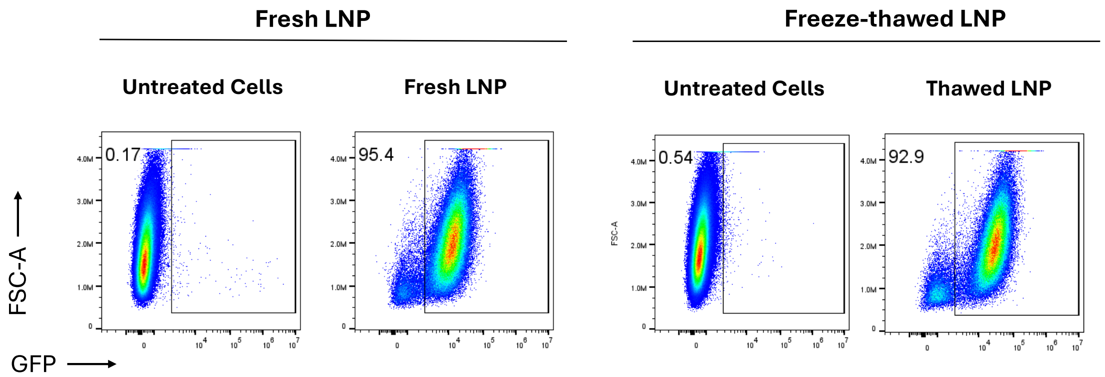

Supplementary Figure 1. Protein expression from HEK293T cells transfected with 1μg mRNA LNPs, either freshly prepared or subjected to two freeze-thaw cycles. The cells were collected for flow cytometry analysis after 24-hours of ALC0315 LNP treatment. The 1μg freshly made ALC0315 LNP produced 95.4% GFP+ cells. After five months of storage and undergoing two freeze- thaw cycles, the same batch of ALC-0315 LNP at 1μg produced 92.9% GFP+ cells at 24 hours post transfection.

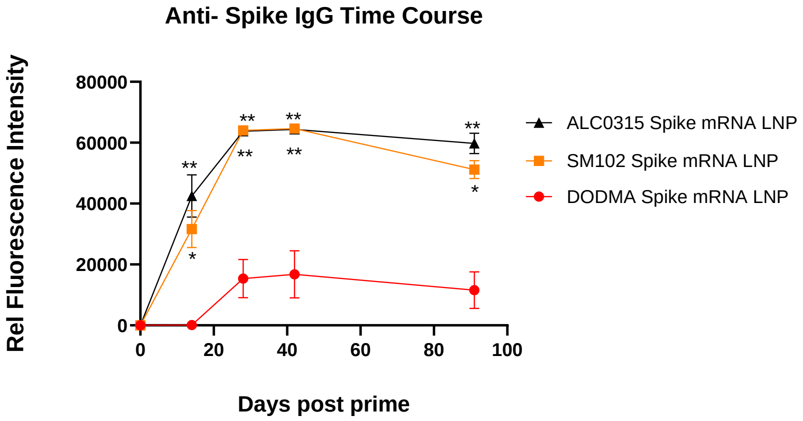

Supplementary Figure 2. Anti-spike IgG time course of top three cationic lipid LNPs. Plasma samples were collected from three mRNA LNP groups at d14, d28, d42 and d91. Samples were assayed for anti-spike IgG response. Statistical analysis was performed between DODMA mRNA LNP group and other groups on each time point. *, p<0.05; **, p<0.01. Top, ALC0315 vs DODMA LNP group; bottom, SM102 vs DODMA LNP group. N=5/group.

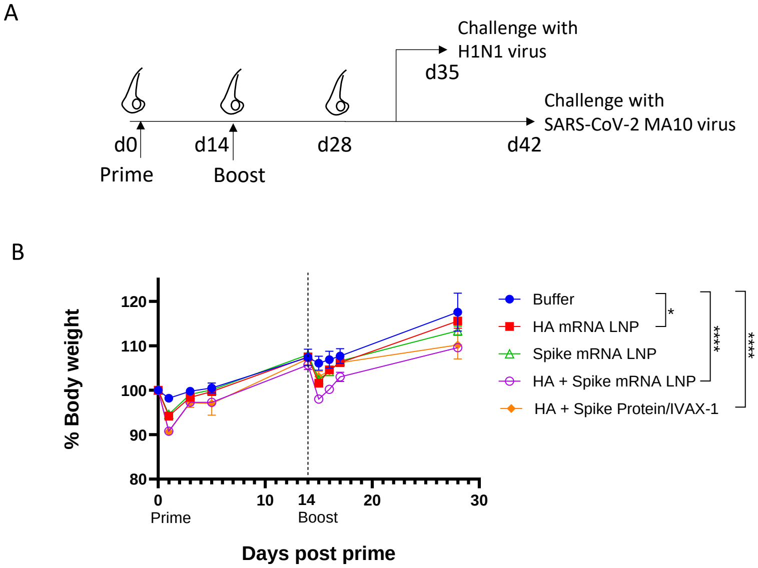

Supplementary Figure 3. Weight change after prime and boost immunizations. Intramuscular immunizations were performed on d0 (prime) and d14 (boost). Weight change was monitored for up to 30 days post prime immunization. Statistical analysis was performed between Buffer group and other groups on 1 day post prime. *, $p<0.05$; **, $p<0.01$. Only significant p values are shown. N=5/group.

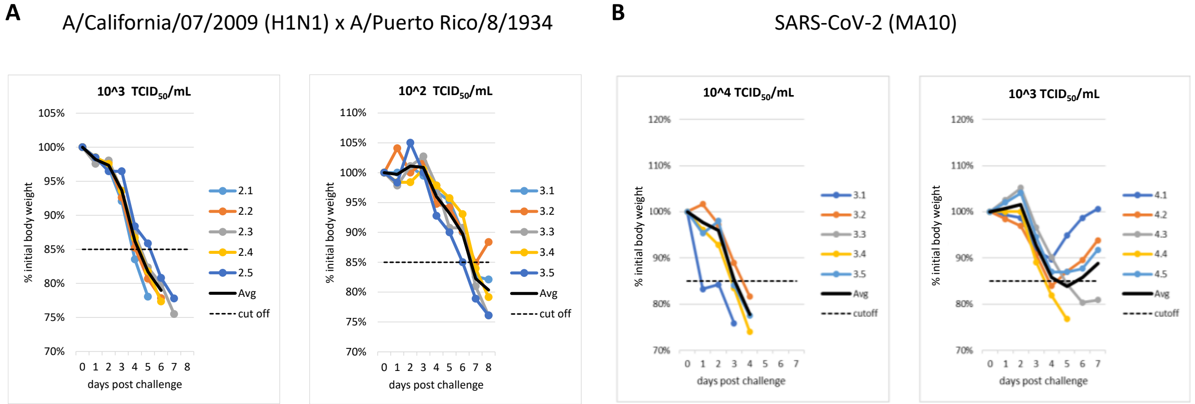

Supplementary Figure 4. Virus dose-ranging study in BALB/c mice. Naive female BALB/c mice were intranasally challenged with 50µL A/California/07/2009 (H1N1)x A/Puerto Rico/8/1934 virus at ABSL2+ (**A**) or SARS-CoV-2 MA10 virus at ABSL3 (**B**). Body weight was measured for up to 8 days post challenge.
